# The antioxidant and neurochemical activity of *Apium graveolens* L. and its ameliorative effect on MPTP-induced Parkinson-like symptoms in mice

**DOI:** 10.1186/s12906-018-2166-0

**Published:** 2018-03-20

**Authors:** Pennapa Chonpathompikunlert, Phetcharat Boonruamkaew, Wanida Sukketsiri, Pilaiwanwadee Hutamekalin, Morakot Sroyraya

**Affiliations:** 1 Expert Centre of Innovative Health Food, Thailand Institute of Scientific and Technological Research, Khlong Luang, Pathumthani 12120 Thailand; 2 Department of Physiology, Faculty of Science, Prince of Songkla University, Hat Yai, Songkhla 90112 Thailand; 3 School of Pharmacy, Walailak University, Thasala, Nakhon Si Thammarat 80161 Thailand; 4 Department of Pharmacology, Faculty of Science, Prince of Songkla University, Hat Yai, Songkhla 90112 Thailand; 5 Department of Anatomy, Faculty of Science, Mahidol University, Ratchathewi, Bangkok, 10400 Thailand; 6 Mahidol University Nakhonsawan Campus, Payuha Kiri, Nakhon Sawan, 60130 Thailand

**Keywords:** *A. graveolens*, MPTP, Tidomet plus, Oxidative stress, Tyrosine hydroxylase, Monoamine oxidase

## Abstract

**Background:**

*Apium graveolens* L. is a traditional Chinese medicine prescribed as a treatment for hypertension, gout, and diabetes. This study aimed to determine the neuroprotective effects of *A. graveolens* extract against a Parkinson’s disease (PD) model induced by 1-methyl-4-phenyl-1,2,3,6-tetrahydropyridine (MPTP) in C57BL/6 mice.

**Methods:**

Male C57BL/6 mice treated with MPTP were orally dosed with *A. graveolens* extract daily for 21 days. Behavioral tests, including a rotarod apparatus, a narrow beam test, a drag test, a grid walk test, a swimming test, and a resting tremor evaluation, were performed. Thereafter, the mice were sacrificed, and monoamine oxidase A and B activity, lipid peroxidation activity, and superoxide anion levels were measured. Immunohistochemical staining of tyrosine hydroxylase was performed to identify dopaminergic neurons.

**Results:**

We found that treatment with *A. graveolens* at dose of 375 mg/kg demonstrated the highest effect and led to significant improvements in behavioral performance, oxidative stress parameters, and monoamine oxidase A and B activity compared with the untreated group (*p* < 0.05). Moreover, the extract increased the number of neurons immunopositive for tyrosine hydroxylase expression compared with MPTP alone or MPTP with a positive control drug (*p* < 0.05).

**Conclusions:**

We speculated that *A. graveolens* ameliorated behavioral performance by mediating neuroprotection against MPTP-induced PD via antioxidant effects, related neurotransmitter pathways and an increase in the number of dopaminergic neurons.

## Background

The prevalence of Parkinson’s disease (PD) increases with age; the disorder affects approximately 1% of the over-60-year-old population worldwide [1, 2] and 4–5% of 85-year-olds [3]. PD is a chronic progressive neurodegenerative disorder characterized by severe loss of dopaminergic neurons in the substantia nigra pars compacta (SNc), leading to dopamine (DA) deprivation in its projections to the striatum and to other neurons in the brainstem, followed by impairment of the neurons in the cerebral cortex responsible for motor processes [4, 5]. The cardinal signs of parkinsonism are resting tremor, cogwheel rigidity, bradykinesia and postural instability [6, 7], which occur when the striatal dopamine depletion was approximately 80% [8]. Several biochemical parameters have been proposed to play key roles in the pathogenesis of PD, one of which is the harmful effect of free radicals and oxidative stress [9] resulting from the crucial role of oxidative stress in inducing mitochondrial complex-1 inhibition and thereby triggering neuronal cell death [10].

One well-accepted and commonplace parkinsonian animal model is generated by intraperitoneal (i.p.) injection of 1-methyl-4-phenyl-1,2,3,6-tetrahydropyridine (MPTP), which is converted by monoamine oxidase type B (MAO-B) to its metabolite 1-methyl-4-phenylpyridinium (MPP^+^) [11]. MPP^+^ exhibits a high affinity for the dopamine transporter (DAT) and is transported into DA neurons, where it impairs respiration by inhibiting mitochondrial complex-1 [12]. This results in increased reactive oxygen species (ROS) production. ROS promotes cell death via oxidatively damaging molecules such as superoxide radicals and hydroxyl radicals and causes lipid and protein peroxidation. Eventually, the affected DA neurons can degenerate by either necrosis or apoptosis [13–15].

To date, efforts to prevent the pathological consequences of PD remain ineffective. While Western remedies fail to treat this complex disease, alternatives such as traditional medicine are attracting interest as a new source of insights. An example of a Western PD treatment limited by side effects is Tidomet Plus, a combination of levodopa and carbidopa. Levodopa is a precursor in the biosynthesis of DA, and its half-life is quite short; therefore, carbidopa, a DOPA decarboxylase inhibitor, is applied to prolong the drug retention time. Still, various undesirable side effects of this drug, namely, drug-induced dyskinesia, akinesia, nausea, stomatitis, hallucinations, psychosis, hypotension, sleep disturbance, anxiety and depression, have been observed [16].

*Apium graveolens* L., a.k.a. celery, is a traditional Chinese medicine prescribed to treat hypertension, gout, and diabetes [17–19]. Its stem, root, and leaf extracts can promote differentiation of neuronal stem cells to neurons and supportive cells such as astrocytes and oligodendrocytes [20]. Apigenin, a compound that can be isolated from celery stems, can promote mature neurons in model systems, both in vitro and in vivo [21]. Moreover, high concentrations of luteolin, another compound found in *A. graveolens*, can inhibit lipopolysaccharide (LPS), which reduces DA recycling and disturb tyrosine hydroxylase (TH) enzyme function in the DA synthesis pathway of neuronal and glial cells in vitro. Apart from these effects, the inhibition of LPS by luteolin can activate supportive cells and expression of tumor necrosis factor-α, nitric oxide and superoxide [22]. Given all the aforementioned effects of *A. graveolens* constituents, we conducted this study to determine the neuroprotective effect of *A. graveolens* extract through its antioxidant effect and related neurotransmitter pathway and investigate whether *A. graveolens* has the capacity to inhibit MPTP neurotoxicity and protect DA neurons.

## Methods

### Chemicals

All chemicals were of analytical grade. Tyrosine hydroxylase (TH) primary antibody, horseradish peroxidase (HRP)-conjugated secondary antibody and a DAB kit were purchased from Thermo Fisher Scientific Inc, Waltham, MA, USA. 1,1,3,3-Tetramethoxypropane (TMP), glutathione peroxidase (GPx), glutathione reductase (GR), reduced L-glutathione, xanthine, xanthine oxidase (XO), and *β*-nicotinamide adenine dinucleotide 2′-phosphate reduced tetrasodium salt were bought from the Sigma Chemical Company, St. Louis, MO, USA.

### Preparation of *A. graveolens* crude extract

Whole *A. graveolens* plants were harvested and dried at Lampang Herb Conservation, Lampang, Thailand, then authenticated by the Forest Herbarium, Bangkok, Thailand (BKF number 188856) and used to prepare a methanolic extract by Dr. Wanida Sukketsiri, Department of Pharmacology, Faculty of Science, Prince of Songkla University, Thailand. The coarse power was exhaustively extracted with 70% methanol (1:1) for 72 h. The methanolic extract was filtrated through Whatman No. 1 filter paper and concentrated under vacuum in a rotary evaporator. Finally, the *A. graveolens* methanolic extract (AGME) was lyophilized with a freeze dryer, placed in a tight container and stored in a cool place until use. All AGME came from the same lot with previous publications [23, 24]. Each vial contained 1 g of dried extract from whole *A. graveolens* plants. The percent yield (*w*/w) of the methanolic extract was 16.0%; characterization of AGME has previously been reported by our group [23, 24]. To guarantee the quality of AGME, HPLC was performed on the methanolic extract and luteoin and apigenin were used as standard markers. Our HPLC result analysis of AGME displayed the active compounds of apigenin 0.031% *w*/w and luteolin 0.030% w/w, respectively compared to standards [24]. The dried extract was freshly prepared throughout the day at the time of each experiment by dissolving it in normal saline solution (NSS) for once-daily oral gavage.

### Animal care conditions

Forty-eight young adult male C57BL/6 mice aged 2 months (25–30 g) were used as experimental models. Sample size was calculated by using Minitab Statistical Software’s Power and Sample Size tools. This strain is very sensitive to MPTP and exhibits Parkinson’s disease (PD)-like symptoms in response [25, 26]. The mice were obtained from the National Laboratory Animal Center, Mahidol University, Salaya, and housed according with the standard guideline of the Southern Animal Unit, Prince of Songkla University. The studies were performed at the Faculty of Science, Prince of Songkla University, Thailand. The mice were randomly separated, five per cage, in a temperature-controlled room (25 ± 2 °C) at 50% relative humidity on a 12/12 h light/dark cycle and had ad libitum access to food and water. All oral administrations in this study were performed once daily between 8.00 and 9.00 a.m. The experimental procedures strictly followed the animal care criteria outlined by the Faculty of Science, Prince of Songkla University (MOE0521.11/582).

### Experimental protocols

The mice were randomly divided into 6 groups. Each group was composed of 8 mice, as follows: in group I, normal control rats received a standard volume of NSS; in group II, PD-like symptoms were induced with MPTP, and the mice received a standard volume of NSS; in group III, PD-like symptoms were introduced with MPTP, and Tidomet Plus (a positive control drug) was administered at 25 mg/kg BW; in groups IV, V, and VI, PD-like symptoms were induced with MPTP, and the mice were treated with AGME at 125, 250 and 375 mg/kg BW, respectively, for 21 days. All groups except the control group were intraperitoneally injected with MPTP-HCl at a dose of 15 mg/kg per day, divided into 4 injections at 2 h intervals on a single day [26]. Mice were tested for motor function and coordination on the rotarod and narrow beam tests (latency time), as well as for balance on the narrow beam walk (foot slip error), drag, and grid walk tests. In addition, the severity of PD was assessed through a swimming and resting tremor scoring at 0, 1, 3, and 7 days after MPTP administration. After the behavioral assays were performed, all of mice were euthanized with pentobarbital-HCl 50 mg/kg, i.p., and the brain areas were separated for neurochemical studies and immunohistochemistry analysis.

### Behavioral tests

#### Rotarod apparatus

The rotarod test for assessing motor and coordination measures the duration of time that mice maintain their balance on a moving rod. Mice were allowed to adjust their posture in order to maintain their balance on a rotating rod at speeds of 5, 10 and 15 rpm three times per day with intertrial intervals of 30 min. The average retention time on the rod was calculated as described previously [27].

#### Challenging narrow beam test

Mice were trained to cross a series of narrow beams (L 100 cm × W 1 cm), elevated at a height of 100 cm from the floor, to reach an enclosed escape platform; latency to reach the platform was used as a measure of motor function and coordination, while the number of foot slip errors was used to assess balance [28].

#### Drag test

The drag test was performed to estimate the ability to balance with the forelimbs in response to a dynamic external stimulus. Mice were gently lifted using the tail and dragged backwards at a consistent speed of approximately 20 cm/s for a fixed distance of 120 cm. The number of touches by each forepaw was counted, and the mean value for the two forepaws was calculated [29].

#### Grid walk test

The grid walking task, which measures foot faults, was used to analyze motor impairments of limb function and foot-placing deficits during locomotion in experimental animals. Mice were placed on a level grid (width 40 cm, length 60 cm) with openings, elevated 100 cm above the floor. Mice without Parkinson-like symptoms typically placed their paws precisely on the frame support their weight while moving along the grid. Each time a paw slipped through an opening in the grid, a “foot fault” was counted [30].

#### Swimming test

A swimming test was carried out to determine motor disability using a round glass swimming tank (length 40 cm, width 25 cm, height 16 cm) filled with water to a depth of 12 cm (maintained at a temperature of 27 ± 2 °C). Mice were scored on the following scale: 0 = no swimming, and the head was above the water; 1 = occasional swim-floating using the hindpaws, 2 = alternation between swim-floating and passively floating, 3 = continuous swimming [31].

#### Resting tremor score

The Parkinson-like symptom of resting tremor was evaluated in the mice. Mice were placed in transparent box that was above the floor of 500 cm, recorded on video for 45 min, and the severity of resting tremor was scored every 3 min on the following scale: 0 = no observation of resting tremor; 1 = minor resting tremor of postural muscles only; 2 = moderate resting tremor sometimes reaching the head; 3 = obvious resting tremor, but not always involving the head; 4 = continuous resting tremor and no movement of limbs or head; and 5 = continuous resting tremor of the whole body [32].

### Neurochemical studies

#### Tissue preparation

After behavioral testing was done, the mice were euthanized, and their brains were quickly removed, cleaned with cold 0.9% NSS and stored at − 80 °C in a freezer. Brain tissue samples were thawed and homogenized with cold 0.1 M phosphate buffer (pH 7.4). The supernatants were prepared to determine lipid peroxidation, % inhibition of superoxide anion (O_2_^−^) and GPx. Moreover, the activity levels of MAO-A and B were also analyzed.

#### MAO-A, B activities

The homogenates were incubated with 500 μM tyramine plus 500 nM pargyline or 2.5 mM tyramine plus 500 nM clorgyline in order to inhibit MAO-B or A activity, respectively. The chromogenic solution prepared in the assay mixture contained vanillic acid (1 mM), 4-aminoantipyrine (500 mM), and peroxidase (4 U ml^− 1^) in potassium phosphate buffer (0.2 M, pH 7.6). The activity levels of MAO-A and B were determined by spectrophotometric measurement according to Holt et al. [33], and the optical density (OD) was measured at 425 nm. The MAO-A and B activity levels are analyzed as units/mg protein, where one unit is determined as 1 μmol of product formed/min.

#### Lipid peroxidation (LPO) activity

Quantitative measurement of malondialdehyde (MDA), which is the product of LPO, was performed according to the method of Ohkawa et al. [34]. A calibration curve was prepared using TMP. A supernatant sample of 0.2 ml was mixed with 1.5 ml of acetic acid (20%) at pH 3.5; 1.5 ml of thiobarbituric acid (0.8%) and 0.2 ml of sodium dodecyl sulphate (8.1%) were added to 0.1 ml of processed tissue samples and then heated at 100 °C for 60 min. Then, the mixture was cooled, and 5 ml of n-butanol-pyridine (15:1) and 1 ml of distilled water were added and vortexed vigorously. After centrifugation at 4000 rpm for 10 min, the organic layer was withdrawn, and its absorbance was measured at 532 nm with a spectrophotometer. The concentration of MDA was expressed as μmol/mg of protein. Total brain protein was quantified by the method of Lowry et al. [35] with slight modification using a Pierce BCA assay kit.

#### Glutathione peroxidase (GPx) assay

GPx catalyzes the reduction of hydrogen peroxide (H_2_O_2_) and lipid peroxides (ROOH), after which glutathione (GSH) reacts with them, resulting in oxidized glutathione (GSSH) and water (H_2_O). Glutathione reductase (GR) catalyzes the reaction of GSSH and nicotinamide adenine dinucleotide phosphate (NADPH), resulting in GSH and NADP^+^. The absorbance of NADP^+^ was measured at 340 nm compared with a blank and a standard curve of GPx concentrations. The values were expressed in units/g of protein [36].

#### Superoxide anion (O_2_^.-^) assay

The O_2_^−^ level was determined by the spectrophotometric procedure described by Ukeda et al. [37], based on the xanthine/XO assay, in which yellow nitro blue tetrazolium (NBT) is converted to blue formazan. Ethylenediaminetetraacetic acid (EDTA), NBT, xanthine and XO added to the reagent mixture, which was then reacted with sample. The absorbance of the formazan chromophore was measured at 560 nm against a blank and a standard curve of 4-hydroxy-2,2,6,6-tetramethyl-1-piperidinyloxy. The data were expressed as % inhibition, which was calculated by the following equation; % inhibition = (A-B)/A × 100 (A = OD of reagent only and B = OD of sample).

### Immunohistochemical staining for tyrosine hydroxylase (TH)

An immunohistochemical study was performed as described by Ahmad et al. [38]. Briefly, the mice were anesthetized with sodium pentobarbital (50 mg/kg i.p.) and then transcardially perfused with 0.9% NSS to clear the blood from the brains. The brain samples were immersed and fixed in 4% paraformaldehyde with 0.1 M phosphate buffer (pH 7.4) overnight at 4 °C, after which they were embedded in paraffin. Coronal paraffin sections 4–5 μm in thickness, selected according to the Atlas of the Mouse Brain [39] to pass through the substantia nigra (bregma 3.16 mm, interaural 0.64 mm), were used for immunohistochemistry study. Each group contained five animals for TH; a rabbit anti-TH polyclonal antibody and a DAB kit (Thermo Fisher Scientific Inc., Waltham, MA USA) were utilized. The paraffin sections were deparaffinized and dehydrated in serial solutions of xylene and alcohol, then washed for 5 min in running tap water and treated with 0.3% hydrogen peroxide in 10% methanol. The sections were then washed three times for 5 min each in 0.01 M PBS, after which they were preincubated for 30 min with 1% glycine. The brain sections were then incubated overnight with anti-TH antibody (1:200) and 0.3% Triton X-100 at room temperature. After a 15-min rinse in 0.01 M PBS, the sections were incubated with biotinylated secondary antibody for 1 h and then with horseradish peroxidase (HRP) complex for 30 min at 37 °C. Immunoreactions were visualized using a DAB kit. The negative control sections were processed in the same way as described above except that the anti-TH antibody was omitted. All the sections were counterstained with hematoxylin, and the slides were dehydrated and cover-slipped. Finally, photomicrographs were taken and analyzed by counting the numbers of positive cells at 200× magnification. The average number of TH-positive cells was used to express the quantity of TH.

### Data analysis

The results are represented as the mean ± S.D. The results of the behavioral analyses, neurochemical measurements and immunohistochemistry analysis were evaluated by one-way analysis of variance (ANOVA) followed by a post hoc multiple comparison analysis (Tukey’s method) using SPSS version 16.0 (SPSS, Cary, NC, USA) and taken as significant only if the *p*-value was less than 0.05.

## Results

Oral administration of the extract from *A. graveolens* did not produce any mortality in mice. No significant difference was detected between the average body weights of treated and control mice (mouse body weights were in the range of 35–40 g). The data are not shown.

### Effect of AGME on the number of forepaw touches during the drag test in MPTP-induced mice

**Fig. 1:**
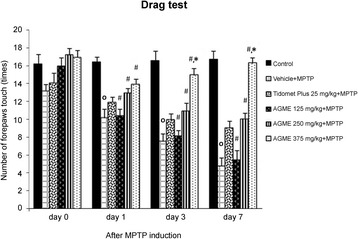
Effect of AGME extract on number of forepaw touches during the drag test at 0, 1, 3, and 7 days after MPTP treatment. Each data column displays the mean ± SD (*n* = 8/group, ^**o**^-*p* < 0.001 compared with the control group; #-*p* < 0.05 compared with the vehicle-treated group; *-*p* < 0.05 compared with the Tidomet Plus-treated group)

The number of forepaw touches in the drag test was determined as a measure of balance ability at 0, 1, 3, and 7 days after MPTP injection (Fig. 1). The significant difference between the control (*n* = 8) and vehicle-treated (*n* = 8) mice (*p* < 0.001) indicated that our PD model was induced successfully. The groups treated with AGME at doses of 250 and 375 mg/kg BW (*n* = 8 each) exhibited a significant rise in forepaw touches compared with the vehicle-treated group at all timepoints (*p* < 0.05). Interestingly, the highest numbers of forepaws touches among all groups (*p* < 0.05) occurred at 3 and 7 days after MPTP treatment in the group given 375 mg/kg BW AGME.

### Effect of AGME on the number of foot faults on the grid walk test in MPTP-treated mice

**Fig. 2:**
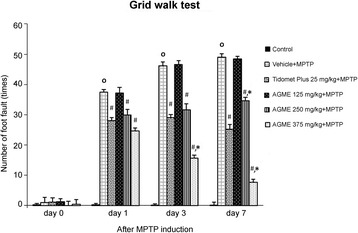
Effect of AGME on the number of foot faults on the grid walk test at 0, 1, 3, and 7 days after MPTP treatment. Each data column displays the mean ± SD (n = 8/group, ^**o**^-*p* < 0.01 compared with the control group; #-*p* < 0.05 compared with the vehicle-treated group; *-p < 0.05 compared with the Tidomet Plus-treated group)

To examine motor and coordination skills, the numbers of foot fault in a grid walk test were examined at 0, 1, 3, and 7 days after MPTP induction (Fig. 2). The group treated with crude extract at doses of 250 and 375 mg/kg BW showed significantly reduced numbers of foot faults compared with the vehicle-treated at all timepoints (*p* < 0.05). Interestingly, at 3 and 7 days after MPTP injection, 375 mg/kg BW AGME clearly improved the number of foot faults compared with the Tidomet Plus and vehicle-treated groups (*p* < 0.05).

### Effect of AGME on latency time and numbers of foot slip errors on the narrow beam test in MPTP-treated mice

**Fig. 3:**
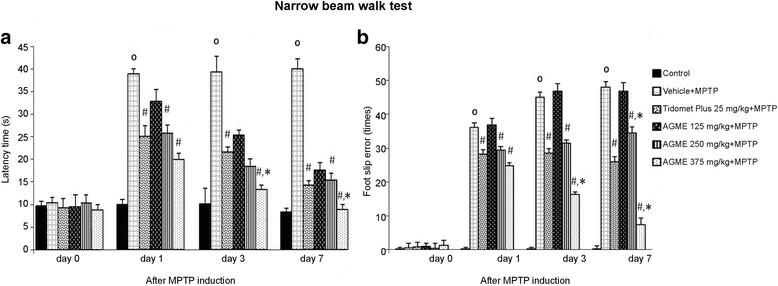
Effect of AGME on latency time (**a**) and foot slip errors (**b**) on the narrow beam test at 0, 1, 3, and 7 days after MPTP induction. Each data column represents the mean ± SD (*n* = 8/group, ^o^-*p* < 0.01 compared with the control group; #-*p* < 0.05 compared with the vehicle-treated group; *-*p* < 0.05 compared with the Tidomet Plus-treated group)

At 0, 1, 3, and 7 days after MPTP injection, the latency time for the narrow beam walk test was analyzed as a measure of motor function and coordination, while the number of foot faults was used to assess balance (Fig. 3). In the vehicle-treated group, the latency time and number of foot errors were significantly larger than those of the control group. The 250 and 375 mg/kg BW extract-treated groups showed significantly reduced latency time and number of foot slip errors compared with the vehicle-treated group at 3 and 7 days after MPTP injection. In particular, 375 mg/kg BW AGME at 7 days of MPTP treatment reduced the number of foot slip errors compared with the vehicle- and Tidomet Plus-treated groups.

### Effect of AGME on resting tremor and swimming scores in MPTP-treated mice

**Fig. 4:**
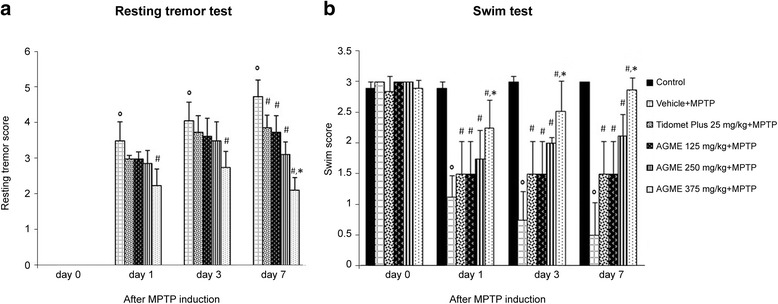
Effect of AGME on resting tremor score (**a**) and swimming score (**b**) at 0, 1, 3, and 7 days after MPTP administration. Each data column represents the mean ± SD (n = 8/group, ^o^-*p* < 0.01 compared with the control group; #-*p* < 0.05 compared with the vehicle-treated group; *-*p* < 0.05 compared with the Tidomet Plus-treated group)

In our PD model, we evaluated the severity of parkinsonian signs through resting tremor and swimming scores at 0, 1, 3 and 7 days after MPTP induction as displayed in Fig. 4. The results demonstrated that, in the MPTP-treated group, the resting tremor and swimming scores became significantly higher than those of the control group. All groups of AGME-treated mice showed dose-dependent improvements compared with the vehicle-MPTP group, and a dose of 375 mg/kg BW provided the largest effect in decreasing both resting tremor and swimming impairment at all timepoints compared with the MPTP-treated group (*p* < 0.05). Note that after 7 days of MPTP administration, the extract at a dose of 375 mg/kg BW showed better efficacy than Tidomet Plus 25 mg/kg (*p* < 0.05).

### Effect of AGME on retention time on the rotarod test in the MPTP model

**Fig. 5:**
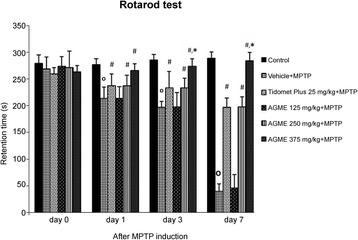
Effect of AGME on retention time on the rotarod apparatus at 0, 1, 3, and 7 days after MPTP administration. Each data column represents the mean ± SD (n = 8/group, ^o^-*p* < 0.001 compared with the control group; #-*p* < 0.05 compared with the vehicle-treated group; *-p < 0.05 compared with the Tidomet Plus-treated group)

To study coordination, we determined the retention time of mice on the rotarod apparatus at 0, 1, 3 and 7 days after MPTP treatment (Fig. 5). The results showed that MPTP treatment increased the retention time significantly. Among all groups, AGME at a dose of 375 mg/kg BW highly decreased retention at all timepoints compared with the MPTP-treated group (*p* < 0.05). In addition, after 7 days of MPTP administration, the extract at a dose of 375 mg/kg BW produced significantly lower retention time than Tidomet Plus 25 mg/kg BW (*p* < 0.05).

### Effect of AGME on MDA level, % inhibition of O_2_^.-^, GPx and MAO-A and B activity in the cerebral cortex and striatum of mice after MPTP treatment

To elucidate the mechanisms of the protective effects of *A. graveolens*, we analyzed oxidative stress parameters such as GPx activity, MDA content and % inhibition of O_2_^.-^ in addition to the activity of MAO-A and B, two components of the related neurotransmitter pathway, in the cortex and striatum of every group of mice. The values of these parameters are represented in Table 1. The MDA content and MAO-A and B activity in the striatum of MPTP-treated mice were significantly increased while the % inhibition of O_2_^.-^ and GPx activity of the vehicle+MPTP treatment group were reduced compared with those of the other groups (*p* < 0.05). However, AGME treatment decreased the MDA level and MAO-A and B activity while increasing % inhibition of O_2_^.-^ and GPx activity; these improvements were observed in mice receiving both doses of AGME (250 and 375 mg/kg BW) after MPTP induction (*p* < 0.05). In addition, treatment with AGME 375 mg/kg BW attenuated the pathological effects of MPTP even more effectively than Tidomet Plus 25 (*p* < 0.05).

**Table 1:** The effect of AGME on MDA level, % inhibition of O_2_^−^, GPx activity, and MAO-A,B activities of cerebral cortex and striatum areas of mice after MPTP treatment

Groups	MDA (μmol/mg protein)		% inhibition of O2-		GPx activity (Unit/g protein)		MAO-A activity (μmol/min.g tissue)		MAO-B activity (μmol/min.g tissue)	
	cortex	striatum	cortex	striatum	cortex	striatum	cortex	striatum	cortex	striatum
Control	2.22 ± 0.26	1.53 ± 0.56	16.48 ± 2.14	19.87 ± 1.99	18.45 ± 1.70	20.87 ± 1.68	3.00 ± 1.01	2.73 ± 1.05	5.01 ± 1.01	4.73 ± 1.05
Vehicle+MPTP	6.34 ± 0.41^a^	5.96 ± 0.22^a^	5.16 ± 1.46^a^	8.32 ± 1.10^a^	5.09 ± 2.27^a^	6.54 ± 2.04^a^	9.02 ± 1.00^a^	10.13 ± 1.18^a^	15.00 ± 1.04^a^	17.13 ± 1.18^a^
Tidomet plus 25 + MPTP	2.81 ± 0.48^b^	1.70 ± 0.13^b^	13.08 ± 1.95^b^	11.87 ± 2.03^b^	15.92 ± 0.81^b^	13.41 ± 1.61^b^	5.46 ± 0.67^b^	6.16 ± 1.25^a^	10.46 ± 0.67^b^	8.16 ± 1.25^b^
AGME 125 + MPTP	2.93 ± 0.41	4.77 ± 0.15	10.85 ± 1.55	9.98 ± 2.55	6.19 ± 1.81	7.83 ± 0.93	8.51 ± 1.19	9.56 ± 1.34	11.51 ± 1.19	10.56 ± 1.34
AGME 250 + MPTP	2.12 ± 0.43^b^	3.75 ± 0.12^b^	12.05 ± 1.99^b^	14.04 ± 2.26^b^	10.55 ± 1.18^b^	12.37 ± 1.15^b^	5.86 ± 0.68^b^	6.70 ± 0.57 ^b^	10.86 ± 0.68^b^	8.70 ± 0.57b
AGME 375 + MPTP	2.01 ± 0.35^b^	0.17 ± 0.19 ^b^	15.75 ± 1.13^b^	18.14 ± 1.26^b,c^	16.54 ± 1.16^b^	18.71 ± 1.26^b,c^	4.21 ± 0.52^b^	3.42 ± 0.72^b,c^	6.21 ± 0.53^b,c^	4.42 ± 0.72^b,c^

Data were represented as mean ± S.D. of three replicates, *n* = 8 each, a vs. control group, *p* < 0.01; b vs. vehicle+MPTP-treated group, *p* < 0.05; c vs.Tidomet plus 25 + MPTP-treated group, *p* < 0.05

### Effect of AGME on the MPTP-induced reduction of TH-positive cell counts in the substantia nigra

**Fig. 6:**
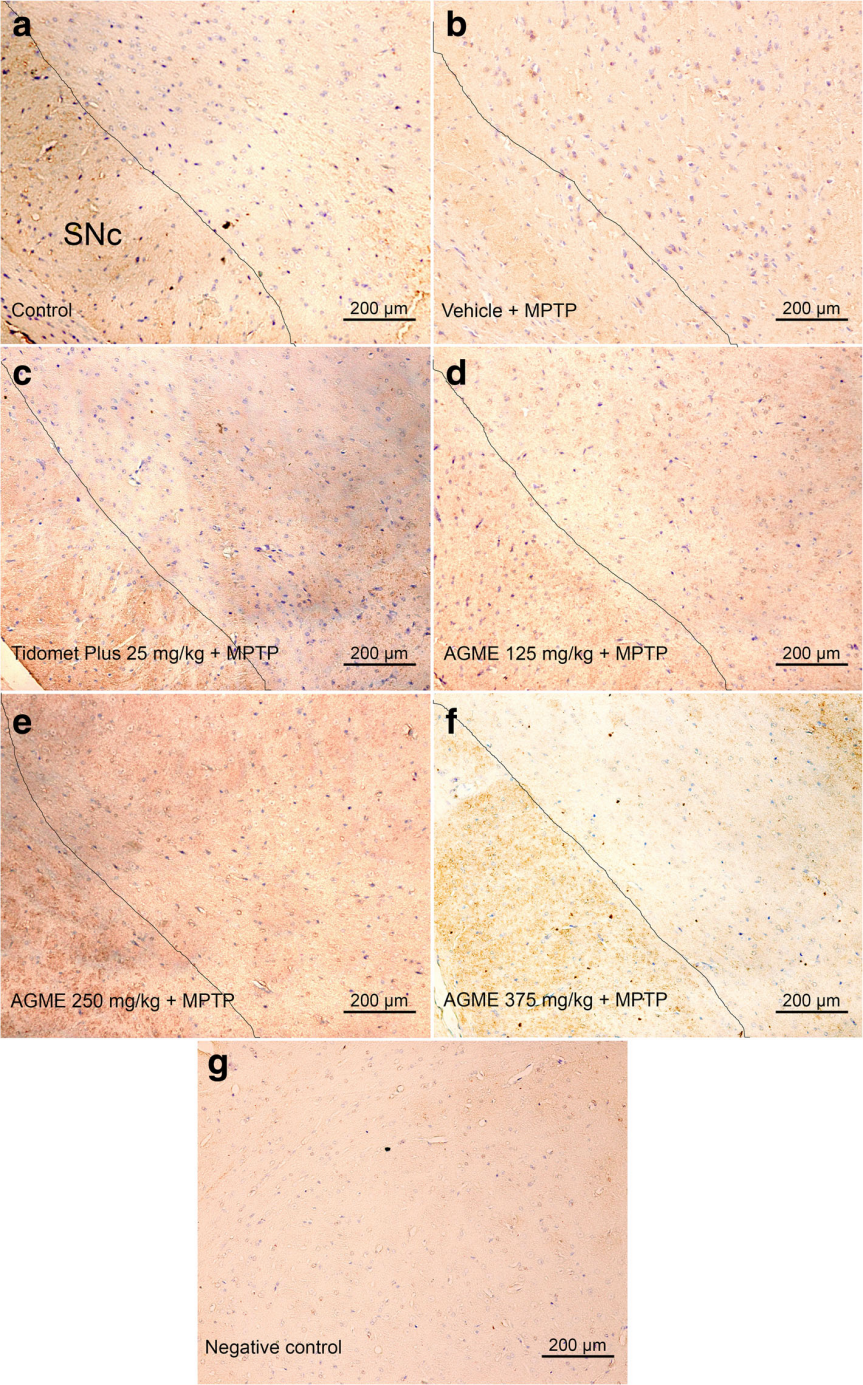
Effect of AGME on the MPTP-induced decrease in TH immunostaining in the substantia nigra of mice. (A) Representative microphotographs showing the control group (**a**), the vehicle (NSS) + MPTP group (**b**), the Tidomet Plus (25 mg/kg BW) + MPTP group (**c**), the AGME (125 mg/kg BW) + MPTP group (**d**), the AGME (250 mg/kg BW) + MPTP group (**e**), the AGME (375 mg/kg BW) + MPTP group (**f**), and a negative control without anti-TH antibody (**g**)

**Fig. 7:**
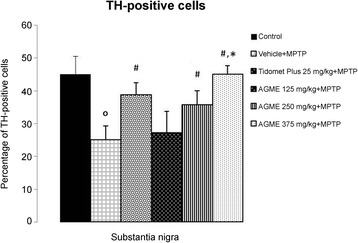
The effect of AGME on the number of TH-immunopositive neurons in the substantia nigra after MPTP administration. The numbers of TH-positive neurons are expressed as the mean ± S.D., (*n* = 4/group), ^o^-*p* < 0.01 compared with the control group; #-*p* < 0.05 compared with the MPTP/vehicle treatment group, *-*p* < 0.05 compared to the Tidomet Plus+MPTP treatment group

TH is the enzyme that represents the rate-limiting step in DA biosynthesis in dopaminergic neurons. In this study, we focused on the number of TH-positive cells in mouse brain slides, especially in the SNc area. Representative microphotographs of TH immunostaining in the substantia nigra are shown in Fig. 6. The TH-positive DA neurons were obvious in the substantia nigra of the control group (Fig. 6a); by contrast, mice that received NSS-only treatment with MPTP administration showed a remarkable depletion of TH-immunopositive cells: the ratio of TH-positive to TH-negative cells in the SNc of those mice was only 25.01 ± 4.26% of the ratio in the control group (Fig. 6b). Interestingly, AGME at the doses of 125, 250 and 375 mg/kg BW ameliorated the ratio of TH-immunopositive cells of MPTP-treated mice, raising the ratio to 27.19 ± 6.16%, 35.73 ± 4.27%, 45.08 ± 4.27% of the control, all higher than the value in the NSS + MPTP-treated group (*p* < 0.05) (Fig. 6d-f). Moreover, AGME at the dose of 375 mg/kg BW produced a higher positive cell ratio than vehicle+MPTP or Tidomet Plus 25 + MPTP (*p* < 0.05) (Fig. 6c), which is consistent with the quantification of TH-positive cells in Fig. 7.

## Discussion

PD is the second most common neurodegenerative disorder and affects many neurons in the nervous system, especially dopaminergic neurons in the midbrain [40]. The severe loss of approximately 50% of DA neurons is an optimal model for drug screening in PD [41]. A strong direct correlation between motor deterioration and DA deprivation in an induced mouse model of PD was also previously reported [31, 42].

Drug therapy of PD with L-DOPA has adverse side effects after a long duration of treatment. These effects consist of dyskinesia, sleep disturbance, and depression [16]; the combination of this drug with other drugs to increase the bio-availability or retention time magnifies this challenge [43]. For example, Tidomet Plus is a combination of levodopa and carbidopa with the purpose of inhibiting peripheral elimination of L-DOPA. Nonetheless, previous research has found that levodopa may be neurotoxic and promote the degeneration of nigrostriatal projections [44]. In addition, prolonged DA treatment is associated with the intracellular accumulation of oxidative stress [45]. To circumvent these drawbacks, the field of herbal medicine has focused on natural products, searching for a low-cost alternative treatment with less severe side effects. *A. graveolens*, especially the well-known variety called Chinese celery, has been used as a spice and a cooked vegetable in the Asia-Pacific region for a long time. The general beneficial effects of this herb are abundant; for example, it is used to treat gout, diabetes, and hypertension [17, 18, 46]. Despite its versatility, the neuroprotective effect of this plant extract in a PD model has not yet been investigated in detail.

We chose the neurotoxin MPTP to specifically and selectively ablate the dopaminergic neurons in the nigrostriatal pathway of a mouse model of parkinsonism because its effects closely imitate the behavioral manifestation and neurochemical mechanisms of PD, including mitochondrial dysfunction, apoptosis, and oxidative stress [47, 48]. In this study, MPTP was administered acutely or subacutely, following the reported method [26], to induce deprivation of DA and its metabolites in the striatum [49].

In addition, its toxic neurological effect is sometimes reversible [50], and experimental animals that receive acute or subacute MPTP do not always show impairment of motor function. Thus, we sought to determine whether treatment of mice with MPTP could induce parkinsonism, using behavioral tests of motor function and coordination (rotarod apparatus and latency to traverse a narrow beam), balance (drag, grid walk, and foot slip errors), and severity of signature signs of parkinsonism (resting tremor and swimming score). Our results suggested that the untreated PD group exhibited significant deterioration of motor function and coordination, balance and severity of parkinsonism compared with the control group that did not receive MPTP (*p* < 0.05), implying that the PD model was successful. Treatment with AGME at doses of 250 and 375 mg/kg BW in PD mice ameliorated all of the behavioral abnormalities found in the vehicle+MPTP induction group (*p* < 0.05). In particular, AGME at a dose of 375 mg/kg BW yielded the highest degree of behavioral improvement, even outperforming Tidomet Plus 25 (*p* < 0.05).

Oxidative stress, including the stress caused by hydroxyl radicals, is one of the key elements of PD pathogenesis; therefore, we measured the MDA level, GPx activity and % inhibition of O_2_^.-^ in the cerebral cortex and striatum, both of which are involved in normal motor function. The results showed that AGME at 250 or 375 mg/kg BW alleviated the effects of MPTP on all measured oxidative stress parameters (*p* < 0.05 for AGME+MPTP vs. NSS + MPTP). Remarkably, the highest dose displayed an even stronger mitigating effect than Tidomet Plus 25 on all parameters of oxidative stress (*p* < 0.05). Taken together, these present results indicate that AGME possesses an antioxidant effect and inhibits oxidative stress pathways, consistent with our previous study, which found that AGME contains high concentrations of flavonoids and phenolic compounds that decrease the total peroxide level and oxidative stress index [23]. In addition, AGME was previously reported to exert an antioxidant effect via reducing the MDA level while elevating GPx activity and the % inhibition of O_2_^.-^ in healthy mice [24, 51].

Generally, enzymatic catabolism of dopamine by the mitochondrial MAO enzymes results in the production of its metabolites and hydrogen peroxide, resulting in high levels of hydroxyl radical deposition through the Fenton reaction [52]. The reduced degradation of dopamine, which can inhibit the MAO-dependent mechanism of dopamine elimination, was our target in this research. Groups treated with AGME at doses of 250 and 375 mg/kg BW-treated groups showed decreases in MAO-A and B activity in both the cortex and the striatum compared with the vehicle+MPTP induction group (*p <* 0.05). Furthermore, the 375 mg/kg BW + MPTP treatment group exhibited significant improvements in MAO-A and B activity over the Tidomet Plus 25 + MPTP-treated group (*p* < 0.05). Therefore, the extract is likely to protect the dopaminergic neurons against inhibition of these enzymes. Based on our study, we propose that AGME possesses the ability to reduce MAO-A activity in association with an anxiolytic effect and an anti-depressant effect in non-lesioned mice [24, 51].

Finally, our immunohistological analysis, presented in Fig. 6, has shown a marked loss of TH immunoreactivity in the SNc 7 days after the acute MPTP injection (15 mg/kg, i.p.) in the AGME-untreated group (Fig. 6b) compared with the control group that did not receive MPTP treatment (Fig. 6a). AGME treatment ranging from 125 to 375 mg/kg BW in a mouse model of Parkinson’s disease gradually increased the abundance of TH-positive cells (Fig. 6d-f) compared with the NSS + MPTP treatment group. These results were also coherent with the quantification of TH-immunopositive cells as represented in Fig. 7.

One further issue needs to be noted here regarding luteolin, an active compound found in *A. graveolens* [53]. Recent works have demonstrated the preventive effect of luteolin against Parkinson’s disease through in vitro experiments [22], and our data corroborate the effect. It is can be speculated that, in an in vivo model, *A. graveolens* possesses neuroprotective activity and contributes to protection against parkinsonism. Interestingly, our previous HPLC data showed that AGME contains 0.030% *w*/w luteolin [24].

## Conclusions

AGME could offer a novel approach to the treatment of Parkinsonism. Our in vivo study demonstrated that the extract is able to ameliorate behavioral impairments, improve oxidative stress parameters, decrease the activity of MAO-A and B, and protect dopaminergic neurons. Our finding establishes this plant extract as a promising candidate for the prevention or treatment of PD. Still, further studies are required to elucidate its molecular mechanism of action in greater detail.

## Abbreviations

DA: Dopamine
MAO-A and B: monoamine oxidase types A and B
MPTP: 1-methyl-4-phenyl-1,2,3,6-tetrahydropyridine
PD: Parkinson disease
SNc: Substantia nigra pars compacta
MPP+: 1-methyl-4-phenyl-pyridinium
DAT: Dopamine transporter
ROS: Reactive oxygen species
OD: Optical density
AGME: *A. graveolens* methanolic extract
HRP: Horseradish peroxidase
LPS: Lipopolysaccharide
TH: Tyrosine hydroxylase
MDA: Malondialdehyde
GPx: Glutathione peroxidase
NSS: Normal saline
GR: Glutathione reductase
XO: Xanthine oxidase
TMP: 1,1,3,3-tetramethoxypropane
O_2_^-^: Superoxide anion
NBT: Nitro blue tetrazolium
i.p.: Intraperitoneal/intraperitoneally
EDTA: Ethylenediaminetetraacetic acid

## References

[1] Eriksen JL, Petrucelli L (2004). Parkinson’s disease-molecular mechanisms of disease. Drug Discov Today Dis Mech.

[2] Samii A, Nutt JG, Ransom BR (2004). Parkinson's disease. Lancet.

[3] Singh N, Pillay V, Choonara YE (2007). Advances in the treatment of Parkinson’s disease. Prog Neurobiol.

[4] Lotharius J, Brundin P (2002). Pathogenesis of Parkinson's disease: dopamine, vesicles and alpha-synuclein. Nat Rev Neurosci.

[5] Dawson TM, Dawson VL (2003). Molecular pathways of neurodegeneration in Parkinson's disease. Science.

[6] Olanow CW, Koller WC (1998). An algorithm (decision tree) for the management of Parkinson's disease: treatment guidelines. American academy of neurology. Neurology.

[7] Obeso JA, Rodriguez-Oroz MC, Rodriguez M, DeLong MR, Olanow CW (2000). Pathophysiology of levodopa-induced dyskinesias in Parkinson's disease: problems with the current model. Ann Neurol.

[8] Betarbet R, Sherer TB, Di Monte DA, Greenamyre JT (2002). Mechanistic approaches to Parkinson's disease pathogenesis. Brain Pathol.

[9] Bolner A, Micciolo R, Bosello O, Nordera GP (2016). A panel of oxidative stress markers in Parkinson's disease. Clin Lab.

[10] McCoy MK, Cookson MR (2012). Mitochondrial quality control and dynamics in Parkinson's disease. Antioxid Redox Signal.

[11] Nagatsu T, Sawada M (2006). Molecular mechanism of the relation of monoamine oxidase B and its inhibitors to Parkinson's disease: possible implications of glial cells. J Neural Transm Suppl.

[12] Boada J, Cutillas B, Roig T, Bermúdez J, Ambrosio S (2000). MPP(+)-induced mitochondrial dysfunction is potentiated by dopamine. Biochem Biophys Res Commun.

[13] Loh KP, Huang SH, De Silva R, Tan H, Benny K, Zhun Zhu Y (2006). Oxidative stress: apoptosis in neuronal injury. Curr Alzheimer Res.

[14] Shibata N, Kobayashi M (2008). The role for oxidative stress in neurodegenerative diseases. Brain Nerve.

[15] Liu Y, Schubert DR (2009). The specificity of neuroprotection by antioxidants. J Biomed Sci.

[16] Borovac JA (2016). Side effects of a dopamine agonist therapy for Parkinson’s disease: a mini-review of clinical pharmacology. Yale J Biol Med.

[17] Covington MB (2001). Traditional chinese medicine in the treatment of diabetes. Diabetes Spectr.

[18] Tong GH, Zhang Y, Zhang YN, Li H, Liu J (2008). Effect of celery seed extract on hyperuricemia in rats. Food Sci.

[19] Syed SF, Rajeev KS (2012). Review on the pharmacognostical & pharmacological characterization of *Apium Graveolens* Linn. IGJPS.

[20] Wen TQ, Lu W, Chen FX, Song HS, Zhao CP, Yu T (2006). *Apium graveolens L.* accelerating differentiation of neural stem cells in *vitro*. J Shanghai Univ.

[21] Taupin P (2009). Apigenin and related compounds stimulate adult neurogenesis. Expert Opin Ther Pat.

[22] Chen HQ, Jin ZY, Wang XJ, Xu XM, Deng L, Zhao JW (2008). Luteolin protects dopaminergic neurons from inflammation-induced injury through inhibition of microglial activation. Neurosci Lett.

[23] Choosri N, Tanasawet S, Chonpathompikunlert P, Sukketsiri W (2017). *Apium Graveolens* extract attenuates adjuvant induced arthritis by reducing oxidative stress. J Food Biochem.

[24] Boonruamkaew P, Sukketsiri W, Panichayupakaranant P, Kaewnam W, Tanasawet S, Tipmanee V, Hutamekalin P, Chonpathompikunlert P (2017). *Apium graveolens* extract influences mood and cognition in healthy mice. J Nat Med.

[25] Feng G, Zhang Z, Bao Q, Zhang Z, Zhou L, Jiang J, Li S (2014). Protective effect of chinonin in MPTP-induced C57BL/6 mouse model of Parkinson’s disease. Biol Pharm Bull.

[26] Sedelis M, Hofele K, Auburger GW, Morgan S, Huston JP, Schwarting RKMPTP (2000). Susceptibility in the mouse: behavioral, neurochemical, and histological analysis of gender and strain differences. Behav Genet.

[27] Rozas G, Guerra MJ, Labandeira-García JL (1997). An automated rotarod method for quantitative drug-free evaluation of overall motor deficits in rat models of parkinsonism. Brain Res Brian Res Protoc.

[28] Pisa M (1998). Regional specialization of motor functions in the rat striatum: implications for the treatment of parkinsonism. Prog Neuro-Psychopharmacol Biol Psychiatry.

[29] Viaro R, Sanchez-Pernaute R, Marti M, Trapella C, Isacson O, Morari M (2008). Nociceptin/orphanin FQ receptor blockade attenuates MPTP-induced parkinsonism. Neurobiol Dis.

[30] Tillerson JL, Miller GW (2003). Grid performance test to measure behavioral impairment in the MPTP-treated-mouse model of parkinsonism. J Neurosci Methods.

[31] Haobam R, Sindhu KM, Chandra G, Mohanakumar KP (2005). Swim-test as a function of motor impairment in MPTP model of Parkinson's disease: a comparative study in two mouse strains. Behav Brain Res.

[32] Lundblad M, Picconi B, Lindgren H, Cenci MA (2004). A model of L-DOPA-induced dyskinesia in 6-hydroxydopamine lesioned mice: relation to motor and cellular parameters of nigrostriatal function. Neurobiol Dis.

[33] Holt A, Sharman DF, Baker GB, Palcic MM (1997). A continuous spectrophotometric assay for monoamine oxidase and related enzymes in tissue homogenates. Anal Biochem.

[34] Ohkawa H, Ohishi N, Yagi K (1979). Assay for lipid peroxides in animal tissues by thiobarbituric acid reaction. Anal Biochem.

[35] Lowry OH, Rosebrough NJ, Farr AL, Randall RJ (1951). Protein measurement with the Folin phenol reagent. J Biol Chem.

[36] Hussain S, WJr S, Ali SF (1995). Age-related changes in antioxidant enzymes, superoxide dismutase, catalase, glutathione peroxidase and glutathione in different regions of mouse brain. Int J Dev Neurosci.

[37] Ukeda H, Maeda S, Ishii T, Sawamura M (1997). Spectrophotometric assay for superoxide dismutase based on tetrazolium salt 3′-{1-[(phenylamino)-carbonyl]-3,4-tetrazolium}-bis(4-methoxy-6-nitro) benzenesulfonic acid hydrate reduction by xanthine-xanthine oxidase. Anal Biochem.

[38] Ahmad M, Saleem S, Ahmad AS, Ansari MA, Yousuf S, Hoda MN, Islam F (2005). Neuroprotective effects of Withania somnifera on 6-hydroxydopamine induced parkinsonism in rats. Hum Exp Toxicol.

[39] Paxinos G, Franklin K (2012). Paxinos and Franklin’s the mouse brain in stereotaxic coordinates. 4^th^ ed. Academic Press.

[40] Dragicevic E, Schiemann J, Liss B (2015). Dopamine midbrain neurons in health and Parkinson's disease: emerging roles of voltage-gated calcium channels and ATP-sensitive potassium channels. Neuroscience.

[41] Ghorayeb I, Fernagut PO, Hervier L, Labattu B, Bioulac B, Tison FA (2002). Single toxin-double lesion' rat model of striatonigral degeneration by intrastriatal 1-methyl-4-phenylpyridinium ion injection: a motor behavioural analysis. Neuroscience.

[42] Henderson JM (2003). Experimental therapeutics of Parkinson's disease. Clin Exp Pharmacol Physiol.

[43] Nagatsua T, Sawadab M (2009). L-dopa therapy for Parkinson's disease: past, present, and future. Parkinsonism Relat Disord.

[44] Fahn S, Sulzer D (2004). Neurodegeneration and neuroprotection in Parkinson disease. NeuroRx.

[45] Caudle WM, Colebrooke RE, Emson PC, Miller GW (2008). Altered vesicular dopamine storage in Parkinson's disease: a premature demise. Trends Neurosci.

[46] Zhang L, Yu WH, Wang C, Zhao F, Qi W, Chan W, Huang Y, SM Wai M, Dong J, T Yew D (2012). DL-3-n-butylphthalide, an anti-oxidant agent, prevents neurological deficits and cerebral injury following stroke per functional analysis, magnetic resonance imaging and histological assessment. Curr Neurovasc Res.

[47] Schmidt N, Ferger B (2001). Neurochemical findings in the MPTP model of Parkinson's disease. J Neural Transm (Vienna).

[48] Schober A (2004). Classic toxin-induced animal models of Parkinson's disease: 6-OHDA and MPTP. Cell Tissue Res.

[49] Miville-Godbout E, Bourque M, Morissette M, Al-Sweidi S, Smith T, Mochizuki A, Senanayake V, Jayasinghe D, Wang L, Goodenowe D, Di Paolo T. Plasmalogen augmentation reverses striatal dopamine loss in MPTP mice. PLoS One. 2016;11. 10.1371/journal.pone.0151020. 10.1371/journal.pone.0151020 PMC4784967 26959819

[50] Franke SK, Kesteren RE, Wubben JA, Hofman S, Paliukhovich I, Van Der Schors RC, Van Nierop P, Smit AB, Philippens IH (2016). Progression and recovery of parkinsonism in a chronic progressive MPTP-induction model in the marmoset without persistent molecular and cellular damage. Neuroscience.

[51] Tanasawet S, Boonruamkaew P, Sukketsiri W, Chonpathompikunlert P (2017). Anxiolytic and free radical scavenging potential of Chinese celery (*Apium graveolens*) extract in mice. Asian Pac J Trop Biomed.

[52] Chiueh CC (1999). Neuroprotective properties of nitric oxide. Ann N Y Acad Sci.

[53] Zhu T, Park HE, Row KH. Purification of luteolin and apigenin from celery leaves using hybrid organic-inorganic monolithic cartridge. J Liq Chromatogr Relat Technol. 2014;37. 10.1080/10826076.2013.825848.

